# Young people’s mental and social distress in times of international crisis: evidence from helpline calls, 2019–2022

**DOI:** 10.1038/s41598-023-39064-y

**Published:** 2023-07-22

**Authors:** Marius Brülhart, Valentin Klotzbücher, Rafael Lalive

**Affiliations:** 1 Faculty of Business and Economics (HEC Lausanne), University of Lausanne, Lausanne, Switzerland; 2 CEPR, London, UK; 3 Institute of Economics, University of Freiburg, Freiburg im Breisgau, Germany

**Keywords:** Psychology and behaviour, Socioeconomic scenarios, Health policy

## Abstract

We document mental and social distress of children, adolescents and adults, using data on 3 million calls to German helplines between January 2019 and May 2022. High-frequency data from crisis helpline logs offer rich information on the evolution of “revealed distress” among the most vulnerable, unaffected by researchers’ study design and framing. Distress of adults, measured by the volume of calls, rose significantly after both the outbreak of the pandemic and the Russian invasion of Ukraine. In contrast, the overall revealed distress of children and adolescents did not increase during those crises. The nature of young people’s concerns, however, changed more strongly than for adults after the COVID-19 outbreak. Consistent with the effects of social distancing, call topics of young people shifted from problems with school and peers to problems with family and mental health. We find the share of severe mental health problems among young people to have increased with a delay, in the second and third year of the pandemic.

## Introduction

Information on psychological distress in the population is a prerequisite for effective crisis management. It contributes to a comprehensive understanding of the costs and benefits of policy measures and helps to identify vulnerable groups in need of targeted support^1–6^.

The COVID-19 pandemic is a case in point: while the general state of physical health and economic activity could be measured fairly accurately and at high frequency^7,8^, population-level information on mental suffering was much sparser. Evidence suggests that mental health problems in the overall population sharply increased around the pandemic outbreak in 2020 and gradually decreased again thereafter^2,9^, with economic aid alleviating some pressure^10^ and the availability of vaccines further reducing distress in the population^11,12^. Less is still known about longer-lasting mental health trajectories, as pandemic fatigue and economic scarring might have set in^13–17^.

A large-scale war such as Russia’s invasion of Ukraine represents another event that could affect mental health well beyond the population immediately affected by the crisis^18^. With the prospect of recurrent natural, economic, and social crises linked to climate change, risks to population mental health look set to remain acute^19–26^.

In this paper, we focus on children and adolescents. It has been argued that lockdowns and associated pandemic mitigation measures weigh particularly hard on the young, for two main reasons. First, the health risks from which those measures are designed to offer protection were much smaller for young people than for the elderly. Second, constraints on social contacts and educational opportunities could be expected to weigh disproportionately on the young.

Survey-based evidence indeed shows children and adolescent mental health to have deteriorated after the outbreak of the pandemic^27^, with girls suffering disproportionately^28^, and school closures being associated with marked negative effects on mental health^29–32^. On the other hand, there is evidence of the pandemic reducing bullying, at school and online^33^. Overall, the evidence is mixed: while children and adolescents reported increased anxiety and depression in surveys, administrative data on suicides, substance abuse and clinical mental health diagnoses rather suggest a decrease in distress during the pandemic^28,34–36^. Some experts have speculated that this apparent paradox may be explained by most administrative data referring to the early months of the pandemic, during which many services were pared back, and that mental stressors, being cumulative over time, will only manifest themselves in “hard” data with a lag^34^. Our long span of high-frequency, large-sample observational data can shed light on this open question.

Most evidence on population mental health is drawn from surveys. In the COVID-19 context, longitudinal studies have been conducted to monitor self-reported mental health and well-being^37–43^. Surveys are a rich and valuable source of information, but they have limitations. First, survey evidence is typically available only with a lag and at a relatively low frequency, as surveys are expensive to implement at regular intervals with an informative sample size^4^. Moreover, survey responses are inevitably sensitive to framing and reporting biases sometimes referred to as “demand effects”^44–48^, sampling issues^49^, and conceptualization challenges^50,51^.

Researchers have therefore tapped complementary data sources to assess population distress at a higher frequency and in user-generated data that are unaffected by study design. The main approaches include analyses of social media sentiment^52–56^, online search behavior^57–61^, and, like this paper, crisis helplines^10,62–68^.

Helplines are proven tools for mental health protection and suicide prevention^69,70^. They were particularly important when lockdown measures restricted social contact and access to regular psychological and social support services^71,72^. Helpline counselors systematically collect anonymous data on caller characteristics and conversation topics. The resulting data provide detailed information on psychological distress in vulnerable populations. Callers to helplines do so on their own initiative, thereby incurring a mental and time cost. In analogy to the concept of *revealed preferences* observed through consumer choices in the field, we therefore consider helpline calls to be a measure of *revealed distress*. On the spectrum of mental health outcomes, helpline data arguably capture a stronger form of distress than survey-based measures, but in contrast to clinical data, they also offer a window on sub-clinical distress.

In this study, we exploit data from the two nationwide German helplines for young people: *Nummer gegen Kummer*, a service aimed specifically at children, adolescents, and their parents, and *TelefonSeelsorge*, Germany’s biggest helpline, aimed at all demographics. We have data on over 3 million phone calls and online conversations between January 2019 and February 2023, allowing us to track the evolution of distress as revealed in calls made by young people before the COVID-19 pandemic, over the course of the pandemic and after the start of the Russia-Ukraine war.

## Results

### Call volumes over time

**Figure 1:**
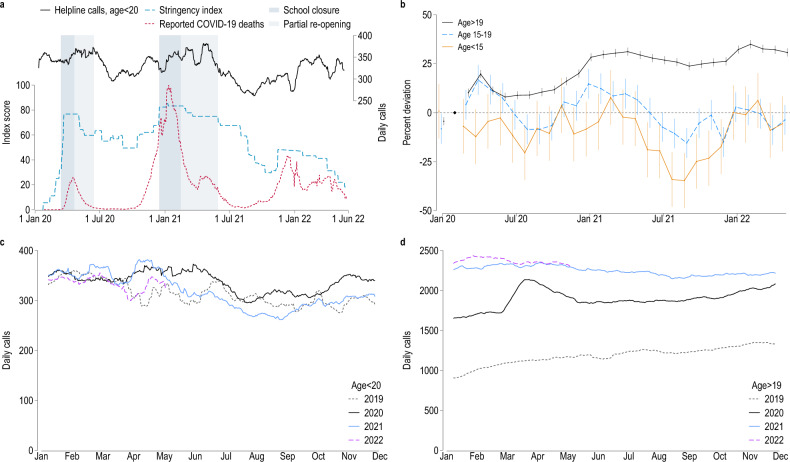
Helpline call volumes, Germany 2019–2022. (**a**) Daily conversations with children and adolescents up to age 19 in black (21-days moving average, right axis), combining data from child and youth helpline *Nummer gegen Kummer* and from general-purpose helpline *TelefonSeelsorge*; newly confirmed deaths related to Covid-19^8^ in red (seven-day moving average, scaled to 896 = 100, left axis); and government response stringency index^73^ in blue (right axis). (**b**) Percent change in daily calls compared to February 2020 by month. Coefficient estimates on week indicators with 95% confidence intervals from a regression where the dependent variable is defined as the natural logarithm of daily calls by age group, see “Methods” Section Eq. 1. (**c**, **d**) Daily call volumes by year and calendar week (21-day moving average) for children and adolescents (**c**) and adult callers (**d**).

The solid black line in Fig. 1a tracks the weekly volume of total helpline calls by children and adolescents from January 2020 to May 2022. This data series includes all calls to the specific children and youth helpline (75% of the overall total of young callers, see Supplementary Table S1 for details) as well as all calls to the general helpline by callers below 20 years of age (25%), and it covers conversations over the telephone (76%) as well as via text-based chat (14%) and email (11%). The dashed red line illustrates the evolution of the pandemic in Germany as measured by COVID-19-related deaths, the dashed blue line shows the stringency of containment measures^8,73^, and the gray areas illustrate periods of general (dark shading) or partial (light shading) school closures. More detailed data series on call and chat volumes, separately by helpline, are shown in Supplementary Figs. S1 and S2.

Figure 1a reveals no strong correlation between (a) the up-and-down of the pandemic and associated containment measures and (b) the consolidated volume of helpline calls by young people. In fact, the volume of calls, while quite volatile week-to-week, is stable across the entire period. The pandemic thus appears to have had no sustained impact on the volume of helpline calls by young people. As can be seen in Supplementary Fig. S1, this trend is not shared equally across the two helplines, with calls by young people to the general-purpose helpline increasing, and calls to the young people’s helpline decreasing slightly. Given that the young people’s helpline is numerically more important, accounting for 78.7% of all calls by Under-20 s (Supplementary Table S1), we can rule out that the volume of helpline calls by children and young people has increased more since the outbreak of the pandemic period than calls by adults.

The strongest apparent regularity is a gradual reduction in the volume of calls after the reopening of schools in 2020 and 2021. School closures, on the other hand, were associated with small increases in call volumes. The observed drop in call volumes after the lifting of school closures could, however, also be a summer effect. This interpretation is confirmed by Fig. 1c, where we overlay the evolutions of our four sample years: a decrease in call volumes by late spring and summer is visible also for the pre-pandemic year 2019.

In Fig. 1b, we show changes in monthly call volumes relative to February 2020, separately by age category. This figure confirms that call volumes by children and adolescents did not systematically exceed the pre-pandemic level, and in some months were even significantly lower. The only indication of a pandemic effect is a statistically significant increase in call volumes by adolescents in the first three months of the pandemic as well as during the second COVID-19 wave of winter 2020–2021.

However, adolescent call volumes reverted to pre-pandemic levels both after the initial three months of the pandemic and after the second wave. Helpline call volumes by children were below pre-pandemic levels in 24 of the 27 pandemic months covered by our data. These patterns contrast strongly with the evolution of call volumes by adult callers: those volumes increased sharply after the outbreak of the pandemic and never reverted to pre-pandemic levels thereafter. The increase in the volume of calls made by adults is also evident from Fig. 1d, which contrasts markedly with Fig. 1c for children and adolescents.

One potential issue with analyses of call volumes is call-answering capacity: unchanged volumes could be consistent with increased demand that could not be met as the supply of counselors did not keep up. Indeed, helplines had to adjust to remote work after the outbreak of the pandemic^10,62^. However, capacity constraints are not a plausible explanation for the sustained stagnation or even reduction in calls that were placed and answered, as shown in Fig. 1. Most helplines were given additional resources to cope with expected increases in demand after the outbreak of the pandemic. Importantly, the increase in recorded adult calls clearly shows that the capacity existed to serve at least part of the increased demand for helpline counseling.

Overall, the evolution of call volumes before and during the pandemic suggests that, while revealed distress noticeably increased among adults, no comparable increase occurred for children and adolescents. This observation runs counter to the often-held view of young people as the main victims of the pandemic and associated mitigation measures. This is broadly consistent with early evidence based on clinical data^29,34,74–76^.

### Conversation topics

Helpline call data provide population-level information not only on the amount but also on the nature of distress. After every call, counselors electronically tick topics from organization-wide pre-defined lists. This generalized procedure results in consistent data on the type of concerns raised by helpline callers.

As we combine data from two large helplines, each with its own topic list, we need to concord them into a harmonized nomenclature. We propose a categorization of 20 topics, each of which contains at least one item from both of the two original topic lists (for details, see Supplementary Table S6). To filter out seasonal fluctuations, we compute mean deviations from the corresponding calendar week of 2019 for every conversation topic (see “Methods” Section, Eq. 2).

**Figure 2:**
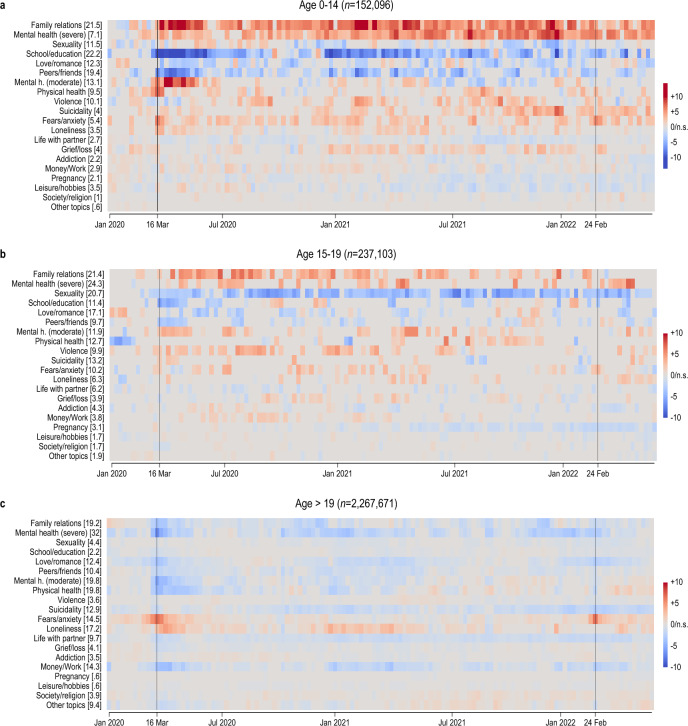
Conversation topics over time—children, adolescents, and adult callers. Deviation of relative topic prevalence from 2019 baseline in percentage points, conversations with child and youth helpline *Nummer gegen Kummer* and with general-purpose helpline *TelefonSeelsorge*. Each row presents coefficient estimates for week indicators from a linear probability model where the dependent variable is equal to one for calls related to a topic and zero otherwise; numbers in brackets report the share of calls in 2019 related to each topic in percent. Separate models for each age group: (**a**) children up to age 14; (**b**) adolescents aged 15–19; and (**c**) adults aged 20 and older. Statistically significant (*P* value < 0.05) increases are shown in red, decreases in blue, and statistically insignificant coefficients as zero/in gray. See “Methods” Section, Eq. 2. Vertical black lines mark the introduction of COVID-19 containment measures in March 2020, and the Russian attack on Ukraine in February 2022.

Figure 2 summarizes the evolution of seasonally corrected harmonized topic prevalence from January 2020 through May 2022, separately for children, adolescents and adults. Changes in helpline-specific, and thus even more detailed, topics are shown in Supplementary Figs. S3 and S4. In Fig. 2, we list topics in decreasing order of their average sample prevalence in 2019 summed across children and adolescents, with numbers in brackets stating the average prevalence for the given age group. Prevalence percentages sum to more than 100% because calls can touch on more than one topic.

Prior to the pandemic, the topics most frequently raised by children were problems with school (22.2%), with family (21.5%), and with peers and friends (19.4%). Adolescents most often sought help for severe mental health problems (depression, self-harm, etc.; 24.2%), family relations (21.4%), and issues with sexuality (20.7%).

Our focus is on changes over time, against the background of the pandemic and the Russian invasion. We note the following, particularly striking observations.

The conversation topics raised by children and adolescents after the outbreak of the pandemic changed more strongly than those raised by adult callers. This is visually evident from a comparison of the three panels of Fig. 2. The mean absolute deviation of weekly percentage-point changes in topic prevalence from January 2020 was 1.79 for children (95% CI 1.71, 1.87, *n* = 2520 coefficient estimates), 1.28 for adolescents (95% CI 1.23, 1.33), and 0.73 for adult callers (95% CI 0.69, 0.76).

For children and adolescents, the pandemic was associated with a significant decrease in the prevalence of calls due to problems with school (most pronounced for children, with − 13.7 p.p. in the week of 15 April 2020, 95% CI − 14.3, − 13.1, detailed numerical estimation results are available in the Figure Source Data), sexuality (most pronounced for adolescents, − 6.8 p.p. in the week of 9 September 2020, 95% CI − 11.1, − 2.5) and peer relationships (− 3.6 p.p. in the week of 18 March 2020, 95% CI − 4.6, − 2.6). These evolutions are consistent with same-age social contacts being reduced during the pandemic, which also limits the potential for relationship problems.

Conversely, the weight of problems within the family markedly increased after the outbreak of the pandemic, both for children (+ 12.0 p.p. in the week of 25 March 2020, 95% CI 9.6, 14.5 and + 14.2 p.p. in the week of 5 Feb. 2021, 95% CI 13.4, 15.0) and for adolescents (+ 7.1 p.p. in the week of 24 June 2020, 95% CI 5.6, 8.7). This and the previous observations together are consistent with social interactions shifting toward the family, away from peers.

Another evident change during the pandemic period is an increase in mental-health-related calls, especially by children but also, with somewhat lesser intensity, by adolescents. In the initial weeks of the pandemic, calls by children related to “moderate” mental-health problems (e.g. stress, boredom) rose sharply (+ 13.1 p.p. in the week of 1 April 2020, 95% CI 10.2, 15.9), but were later superseded by an increase in calls due to mental health problems that we class as severe (e.g. depression, self-harm, + 10.9 p.p. in the last week of 2021, 95% CI 10.1, 11.8). This is indicative of a certain segment of children callers suffering from increasingly serious mental health issues as the pandemic lasted beyond the initial wave. For both children and adolescents, severe mental health issues peaked in the winter of 2021–2022, consistent with pandemic fatigue/exhaustion.

The evolution of topics raised by adult callers is very different from that of younger callers. Calls by adults mainly increased due to fears and anxiety (including fear of infection (+ 6.7 p.p. in the week of 18 March 2020, 95% CI 6.6, 6.9) and due to loneliness (+ 4.4 p.p. in the week of 6 April 2020, 95% CI 4.20, 4.6). This observation is consistent with patterns found internationally^10^. Fears by adults spiked around the outbreak of the pandemic in spring 2020, and again around the Russian invasion of Ukraine in spring 2022.

Young callers also differ from adult callers in terms of what might be considered the most serious topics, violence and suicidality. Among adult callers, the prevalence of those topics has fallen below its pre-pandemic level throughout our observation period. In contrast, we observe increases in the prevalence of calls linked to violence for both children (+ 5.5 p.p. in the week of 16 September 2020, 95% CI 4.0, 7.1) and adolescents (+ 5.0 p.p. in the first week of 2021, 95% CI 2.29, 7.68). For children, we moreover detect an increase in the share of calls related to suicidality, even though this is from a low base (4% of calls). The share of suicide-related calls by adolescents, however, remained roughly unchanged relative to before the pandemic.

When we split the data by caller gender, we find that the share of calls made by girls increased during the pandemic (Supplementary Table S5 and Fig. S5). Among children, the share of girls increased from 50% in 2019 to 56% in 2021 and remained high (54%) in 2022. The increase was even stronger among adolescents, where the share of calls by females rose from 52 to 58% between 2019 and 2021, and remained high (57%) in 2022. In contrast, the gender composition of callers remained stable among adults (67% in 2019, 68% in 2021, and 67% in 2022).

The increase in severe mental health issues is mostly driven by girls (up to + 15.2 p.p. for girls younger than 15 during the last week of 2021 and + 7.8 p.p. for adolescent girls of age 15–19 in the week of 23 April 2022, 95% CI 7.7, 22.7 and 1.4, 14.1, respectively; compared to a maximum increase of + 3.4 p.p. for boys below the age of 15 in the week of 28 May 2021, and + 5 p.p. for boys aged 15–19 in the week of 2 July 2021, 95% CI 1.1, 5.6 and 2.7, 7.2, respectively), see Supplementary Figures S7–S8). Girls and young women appear to have been particularly hard hit by the pandemic and associated restrictions.

### Parent helpline

Most children and adolescents live with their parents. This implies (a) that parents can offer a complementary source of information on the distress and mental health of young people, and (b) that the well-being of children and adolescents has strong externalities, as it immediately affects their parents. Data from a parent helpline allow us to address these two dimensions: an alternative angle on the well-being of children as viewed by their parents, and insights into problems linked to parents’ responsibilities for their children.

We observe a sharp and sustained increase in calls to the parent helpline after the outbreak of the pandemic contrasts with the largely unchanged volume of calls by children and adolescents themselves (Supplementary Fig. S1a). The surge in call volumes in 2020, however, is likely to have been driven at least in part by supply rather than by demand, as that helpline was publicized more actively and operating hours were extended after the outbreak of the pandemic^77^, see “Methods” Section for details.

**Figure 3:**
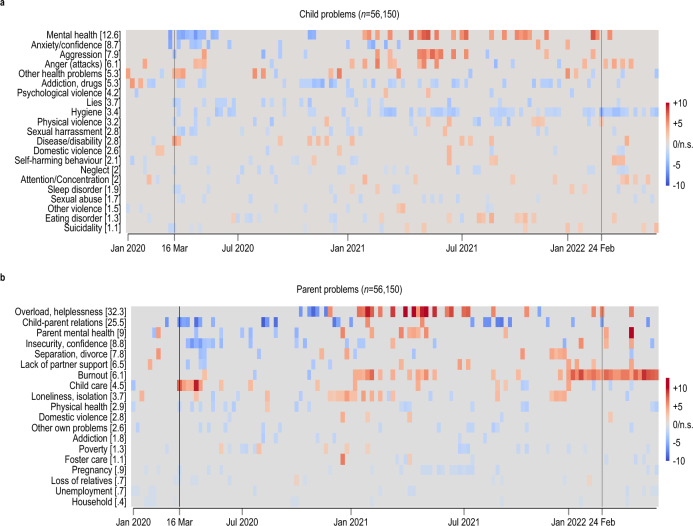
Conversation topics over time—Parent helpline Deviation of relative topic prevalence from 2019 baseline in percentage points, conversations with *Nummer gegen Kummer* helpline targeted at supporting parents. Each row presents coefficient estimates for week indicators from a linear probability model where the dependent variable is equal to one for calls related to a topic and zero otherwise; numbers in brackets report the share of calls in 2019 related to each topic in percent. See “Methods” Section, Eq. 2. (**a**) Conversation topics related to problems of children; b, topics related to parents’/callers’ own problems. Statistically significant (*P* value < 0.05) increases are shown in red, decreases in blue, and statistically insignificant coefficients as zero/in gray. Vertical black lines mark the introduction of lockdown measures following the COVID-19 pandemic outbreak on 16 March 2020, and the Russian invasion of Ukraine on 24 February 2022.

Supply-side factors, however, cannot affect changes over time in the relative prevalence of conversation topics. We illustrate the evolution of topic prevalence in Fig. 3, analogous to Fig. 2. Here, we do not need to harmonize topic nomenclatures and can instead take them as designed by the helpline.

In Fig. 3a, we present topics related to callers’ children (see Supplementary Figure S9 for a decomposition into age groups 0–14 and > 15). The most striking pattern is an initial decrease of calls related to child mental health, which is the topic raised most frequently by parents (− 6.4 p.p. in the week of 22 April 2020, 95% CI − 9.4, − 3.3 ). That decrease, however, was followed in 2021 by a marked increase (+ 7.5 p.p. in the week of 7 May 2021, 95% CI 2.7, 12.2). This evolution is consistent with the pandemic initially alleviating mental-health problems but increasing them as it dragged on after about a year, confirming the patterns observed in calls from children and adolescents themselves.

A similar delayed increase in distress is evident in parents’ problems with their own lives (Fig. 3b): the topic “overload, helplessness” sharply increased in the second year of the pandemic (+ 10.7 p.p. in the week of 16 April 2021, 95% CI 4.9, 16.5), whereas the strongest increase in the prevalence of calls due to “burnout” only appeared in the third year of the pandemic (+ 9.4 p.p. in the week of 7 May 2022, 95% CI 5.6, 13.1). Problems linked to “child care”, in contrast, spiked in the early months of the pandemic (+ 9.7 p.p. in the week of 15 April 2020, 95% CI 6.7, 12.8) but returned to pre-pandemic levels thereafter, despite renewed school closures during the second COVID-19 wave in Germany (see Fig. 1a).

Overall, more calls to the parent helpline concern problems of the parents themselves (73%) than problems of their children (53%, see Supplementary Table S4). The share of parent-related calls increased by 4.1 percentage points between 2019 and 2022, and that of child-related calls to the parent helpline increased by 2.4 percentage points. This suggests that the pandemic was associated with a somewhat stronger increase in parents’ own problems.

Overall, we read the evidence from the parent helpline as suggesting that the toll of the pandemic weighed mainly on the parents themselves and grew gradually more severe as the pandemic lasted. This mirrors the evolution towards more severe mental health issues observed in children’s and adolescents’ own calls and is thus consistent with significant intra-family externalities.

### War and pandemic

The data allow us to compare the mental and social effects of two large-scale crises: the outbreak of the COVID-19 pandemic in early 2020, with direct ramifications on everyday life, and the Russian attack on Ukraine in early 2022, which did not affect daily life immediately outside Ukraine, Russia and neighboring countries. Call volumes by children and adolescents did not noticeably increase in the wake of the Russian invasion (see Fig. 1). Calls by adults increased slightly in February 2022 (from an average of 2350 calls per day in January to 2417 in February), but most of that increase occurred prior to the invasion on February 24. This is in contrast to the pronounced increase observed after the outbreak of the pandemic (from 1703 average calls per day in February to 1897 in March, and 2080 in April 2020) or during the second COVID-19 wave (from, on average, 1915 calls per day during October 2020 to 2262 daily calls in January 2021). Judging by those data, both the war and the pandemic have affected adults more than the young.

**Figure 4:**
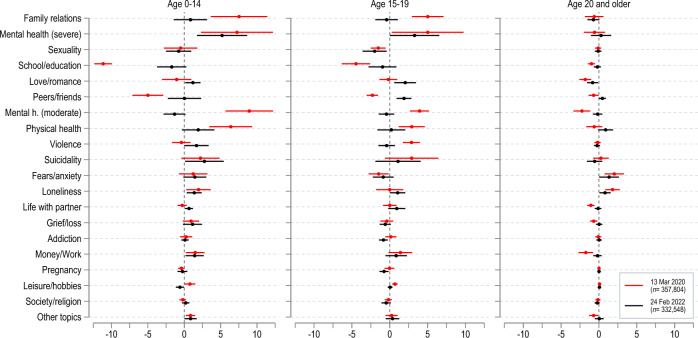
Conversation topics after pandemic outbreak and Russian invasion. Estimated coefficients from separate linear probability models with 95% confidence intervals. Short-term change in topic prevalence following the pandemic outbreak (difference between 13 March–13 April 2020 and 1 January–13 March 2020, compared to the change over the same time in 2019) in red; and the Russian invasion of Ukraine (difference between 24 February–24 March 2022 and 1 January–24 March 2022, compared to 2019) in black. See “Methods” Section, Eq. 3.

In this context too, we can look beyond call volumes and search for patterns in the nature of calls. In Fig. 4, we compare changes in topic prevalence in the first month of the pandemic and in the first month of the war, in both cases scaled relative to the topic prevalence of the relevant age group in 2019.

We find that calls due to fears and anxiety increased in the wake of both the pandemic and the war. This effect is statistically significant and of very similar magnitude across the two crises for both children (+ 3 p.p. after the pandemic outbreak, 95% CI 1.3, 4.7; and + 2.8 p.p. after the invasion of Ukraine, 95% CI 1.1, 4.55) and adults (+ 4 p.p. after the pandemic outbreak, 95% CI 3.0, 4.9; and + 2.7 p.p. after the invasion of Ukraine, 95% CI 1.4, 4.0). Adolescents, however, were different: at the start of neither of the two crises did they call more often out of fear or anxiety (+ 0.4 p.p. after the pandemic outbreak, 95% CI -0.7, 1.5; and + 0.5 p.p. after the invasion of Ukraine, 95% CI − 1.0, 2.1).

Some differences between the two crises also emerge. Family-related problems increased (+ 5.8 p.p. for children and + 3.2 p.p. for adolescents, 95% CI 2.1, 9.5 and 1.0, 5.4, respectively), and school-related and peer-related problems decreased significantly in the early weeks of the pandemic, both for children (− 11.1 and − 6.0 p.p., 95% CI − 12.2, − 9.9 and − 8.1, − 3.8, respectively) and adolescents (− 4.4 and − 3.3 p.p., 95% CI − 6.2, − 2.5 and − 4.1, − 2.6, respectively). Those issues do not seem to have been affected by the outbreak of the war. This difference is easy to rationalize with the school closures and lockdown of early 2020.

For adults, the increase in calls out of fear and anxiety was similar after the outbreak of the pandemic (+ 4.0 p.p., 95% CI 3.0, 4.9) and after the outbreak of the war (+ 2.7 p.p., 95% CI 1.4, 4.0). However, calls due to loneliness increased only after the outbreak of the pandemic (+ 2.9 p.p., 95% CI 1.8, 3.9) but not after the outbreak of the war (− 0.4 p.p., 95% CI − 0.9, 0.0).

In summary, our analysis suggests that COVID-19 was the bigger of the two shocks when measured by the volume of calls made by adults. Neither of the two crises was followed by a sustained change in the volume of overall helpline calls placed by children and adolescents. Differences in the nature of calls during the two crises, in terms of the relative prevalence of conversation topics, have a straightforward explanation in the unequal nature of those crises (lockdown and school closures early in the pandemic but not in the war) and therefore validate the informativeness of helpline call data.

## Discussion

We analyze calls to crisis helplines as a way to monitor trends in revealed distress by children, adolescents, and adults, over the course of the COVID-19 pandemic that had started in early 2020 and after the Russian invasion of Ukraine in February 2022. Data collected routinely by crisis helpline counselors provide a detailed and high-frequency measure of the prevalence of distress, as measured by call volumes, and of its nature, as measured by call topics, across different segments of the population.

We find that the volume of calls by children and adolescents increased less in the wake of either of those international crises than calls by adults. To the extent that call volumes reflect the level of social and mental distress in the population, this suggests that children and adolescents were less impacted by those crises than adults. It seems plausible to attribute this finding at least partly to different affectedness: adults faced greater health risks from COVID-19 and are more likely to live alone. They also were more likely to be informed about the wider security concerns after the Russian attack on Ukraine. Our data show that the increase in adult call volumes was mainly driven by fear (including fear of infection) and by loneliness.

The composition of calls by young people, however, changed differently from that of calls by adults. Since the outbreak of the pandemic, we observe a decrease in young people raising problems at school and with peers and an increase in young callers talking about problems with family and mental health. The mental health issues appear to have become more severe over the course of the pandemic. This pattern too has a natural explanation, as school closures and social distancing reduced opportunities for peer contact and forced closer contact with family members.

In short, our analysis suggests that the overall level of distress among young people as well as among adults (net of the proximate effects of the pandemic) has remained roughly unchanged, even if some subgroups of the population (e.g. girls) were more strongly affected.

Analyses of administrative data are consistent with no or small effects of the pandemic on mental health. For instance, suicide rates do not appear to have been significantly affected by the pandemic^9,74–76,78^ and hospital admissions for mental health issues have been flat or decreasing^79^. Police reports of child maltreatment and domestic violence did not increase substantially after the onset of the pandemic^80,81^.

Survey evidence, however, has on the whole pointed to a significantly raised level of distress throughout the pandemic, in clear contrast to clinical data^34^. Numerous surveys have reported increases in mental health problems after the outbreak of the pandemic^2,37,82–84^. Surveys targeted at children and adolescents found similar increases^27,28^. Those survey findings match our observations that, on aggregate, girls suffered more distress than boys and that for the affected subpopulation the severity of mental health issues increased over time.

One interpretation of the apparent differences is that it is difficult for surveys to cover the whole range of possible problems. Hence, they may struggle to uncover potential “offsetting” trends in problems other than those queried by the investigator. As a result of this issue, and also due to the difficulty of weighting different problem types, it is challenging to aggregate survey items into a combined measure of total distress. Helpline data allow us to circumvent this aggregation problem by considering call volumes, which provide an indication of the number of people for whom the level of distress exceeded the time and inhibition threshold for calling a helpline, regardless of the nature of the problem. This, in addition to offering a measure of revealed distress independent from potential experimenter demand effects, is a key advantage of helpline data.

Our analysis offers numerous avenues for further research. Child and youth helplines of other countries could be analyzed. If multiple countries, or regions within a country, could be covered, then panel data econometric techniques could be applied to disentangle the effects of different overlapping causes^10^. Another, even more challenging, objective could be to characterize the composition and representativeness of helpline caller populations. Such an analysis would require close collaboration with helpline managers as well as a solution to extract the required information without compromising caller anonymity.

## Methods

### Helpline statistics

We received data on calls with the two largest crisis helpline services in Germany, collected since January 2019. For calls that develop into conversations, counselors fill in an electronic report on individual caller characteristics and issues discussed, selecting relevant items from a pre-defined, helpline-specific list of topics. The helplines guarantee anonymity to their callers, and it is impossible to identify callers from the conversation-level data. Callers are informed that anonymous call data are collected for reporting and statistical purposes, explicitly in the terms and conditions and implicitly in annual reports and online publications.

Two of the helplines analyzed are operated by *Nummer gegen Kummer e.V.* (NgK), a non-profit organization established in 1980 that coordinates dedicated helpline services for children and adolescents–the *Kinder- und Jugendtelefon* (KJT) phone service and the chat/email-based *Online-Beratung* (OB)—as well as a telephone helpline for parents, the *Elterntelefon* (ET). NgK services have been supported by Deutsche Telekom AG since 1991, with additional funding provided by the German Federal Ministry for Family Affairs, Senior Citizens, Women and Youth, as well as by the European Union and other donors. The KJT service is free of charge, currently provided by 77 centers across Germany, where contacts are answered from Monday to Saturday between 2 and 8 pm by around 3200 trained volunteer counselors. The OB support via live chat is available on Wednesdays and Thursdays from 2 to 6 pm, E-mails are usually answered within 1–2 days. Data on chats are collected only from January 2020 onwards; for 2019 the OB data cover only E-mail contacts. The dedicated ET parent helpline has been operational nationwide since 2001 and currently comprises a network of 38 helpline centers. The minimum operating hours across all centers are Monday–Friday between 9 and 11 am, as well as Tuesdays and Thursdays from 5 to 7 pm. As the service gained popularity after the outbreak of the COVID-19 pandemic, ET operating hours were extended to cover Monday–Friday 9 am to 5 pm, and additionally from 9 am to 7 pm on Tuesdays and Thursdays. Further details are available on the NgK website at www.nummergegenkummer.de.

The third helpline analyzed, *TelefonSeelsorge e.V.* (TS) is the largest service in Germany, providing around-the-clock free general-purpose support by telephone, mail and online chat. The helpline is run by the Protestant and the Catholic churches and supported by the German Federal Ministry for Family Affairs, Senior Citizens, Women and Youth, as well as Deutsche Telekom AG. Of the over 100 active local centers, 82 started to report information about conversations in 2019; 6 additional centers started to collect data in January 2020. For additional information, see www.telefonseelsorge.de. Supplementary Tables S2–S4 present descriptive statistics.

### Call volumes

For our main analyses, we combine data from the NgK child helpline and the general-purpose TS (both phone and chat/mail services, see Supplementary Table S1), exploiting the available information on caller characteristics to consistently distinguish sub-groups by age and gender. After every conversation, counselors record information on caller’s sex and age, as stated during the conversation or as inferred by the counselor. We distinguish three age groups: *children* up to age 14, *adolescents* aged 15–19, and *adults* aged 20 and older. In the Supplementary Information we show details separately for the two helpline services: Supplementary Fig. S1 shows the evolution of conversation volumes over time, Supplementary Fig. S2 shows the distribution of caller age.

Figure 1 shows the development of daily call volumes across age groups. Panel A shows the time series of daily calls identified as coming from young callers, between January 2020 and May 2022. Panel B shows the results of a regression specified as in Eq. 1, where the dependent variable is defined as the natural logarithm of the number of calls reported for day $t$:

1 $$\log \left( {Calls_{t} } \right) = \gamma_{1} \,Jan2020_{t}^{1} + \mathop \sum \limits_{\tau = 1}^{27} \gamma_{\tau } \,Month_{t}^{\tau } + \theta_{t} + \epsilon_{t}$$

The indicator variable $Jan\,2020_{t}$ is equal to one for days in January 2020 and zero otherwise. The variables $Month_{t}$ denote the running month $\tau$, counting from March 2020 ($Month_{t}^{1}$), so that the coefficients $\gamma$ can be interpreted as the percentage deviation in monthly call volumes compared to February 2020. $\theta_{t}$ denotes weekday indicators that account for variation through lower capacity at the KJT on weekends. We estimate separate regressions for each of the three age groups.

### Deviation in conversation topic prevalence

For the analysis of conversation topics in Fig. 2, we drop 227,453 calls for which no information on the age of callers is available, as well as 41,683 calls without information on at least one conversation topic. This leaves us with a sample of 2,654,160 calls. To summarize the development across different helpline services, we have built a list of 20 topics each of which covers topics as defined in either helpline-specific nomenclature. Supplementary Table S6 shows the mapping of helpline-specific topics to our common categorization of non-exclusive topics; Supplementary Table S7 lists the original German descriptions of the detailed helpline-specific topics.

To capture deviations in the relative prevalence of conversation topics while controlling for seasonal patterns, we use the conversation-level data and estimate separate linear probability models for each of the three age groups. Specifically, we estimate Eq. 2, where the dependent variable $T_{i,t}$ is set equal to one if topic $T$ has come up in conversation $i$ that took place in week $t$, and zero otherwise:

2 $$T_{i,t} = \mathop \sum \limits_{\tau = 53}^{178} \left[ {\delta_{\tau } Week_{t}^{\tau } } \right] + \mu_{t} + \xi_{j\left( i \right)} + \epsilon_{i,t}$$

The indicator variables $Week_{t}^{\tau }$ for the running week $\tau$, counting from the first week of 2019, are equal to one for calls during the respective week, and zero otherwise. Using the year 2019 as a reference, we include indicator variables from week 53 (January 2020) onwards. Calendar month-indicators $\mu_{t}$ control for seasonal regularities, and helpline-specific intercepts $\xi_{j\left( i \right)}$ capture time-invariant differences across helplines, where $j\left( i \right)$ identifies the helpline which answered call $i$. Estimates of the coefficients $\delta_{\tau }$ can be interpreted as percentage-point deviations in relative prevalence as compared to calls to the same helpline in the corresponding calendar month of 2019. Standard errors are clustered at the helpline-month level.

Due to a technical problem at the TS helpline, information on conversation topics was recorded only for a relatively small share of contacts during the second week of September 2021. For the illustration in Fig. 2, we display instead the deviation for this week through interpolation based on the average coefficient estimates for the previous and subsequent week.

Using the same methodology, we obtain changes in topic prevalence from separate regressions separately for female and male callers. Calls for which information on caller sex is unavailable are dropped from the sample (18,117 calls), as well as 3317 callers labeled “diverse”. Results are shown in the Supplementary Figs. S6–S8. Supplementary Figs. S3 and S4 present results for the full list of topics at the helplines separately.

Figure 3 shows the results obtain for the 56,150 calls recorded at the NgK ET parent line between 1 January 2019 and 31 May 2022. Supplementary Table S4 shows descriptive statistics. Using the information on the age of children that adult callers refer to during calls, we repeat the analysis splitting the sample according to whether callers talk about children below the age of 15 or adolescents older than 14. These results are shown in Supplementary Fig. S9. Here, we drop 7464 calls for which no information on children’s age is available. Of the remaining 48,686 calls, 3570 (6.4%) refer to both children and adolescents. 2057 (3.7%) of the calls to the parent helpline do not refer to children of either age group.

### Short-run changes after pandemic outbreak and Russian invasion

The results presented in Fig. 4 are based on separate regressions using data on calls around the two crisis events to isolate short-term changes in topic prevalence.

For the pandemic outbreak, we use data covering the time from 1 January 2019 to 13 April 2019, and the same time in 2020. We define the indicator variable $Post\,\,out\,break_{t}$ set to one for calls on/after 13 March 2020, when the first significant lockdown measures were enacted^73^, and we estimate linear probability models for every topic according to Eq. 3:

3 $$T_{i,t} = \gamma Post\,out\,break_{t} \times \alpha_{a\left( i \right)} + \vartheta_{w\left( t \right)} + \Theta_{y\left( t \right)} + \xi_{j\left( i \right)} + \alpha_{a\left( i \right)} + \epsilon_{i,t} .$$

We include week-of-year indicators $\vartheta_{w\left( t \right)}$, where $w\left( t \right)$ identifies the week-of-year of calendar time $t$ to capture seasonal trends, and indicators for the year $\Theta_{y} \left( t \right)$, where $y\left( t \right)$ identifies the year of calendar time $t$, and helpline indicators $\xi_{j\left( i \right)}$ and age-group $\alpha_{a\left( i \right)}$, where $a\left( i \right)$ identifies the age group of the person who placed call $i$. Estimates of the coefficient $\gamma$ quantify the age-specific change in topic prevalence during the time from 13 March 2020 to 13 April 2020, compared to the time from 1 January 2020 to 12 March 2020, relative to the change observed between the periods 13 March 2019 to 13 April 2019 and 1 January 2019 to 12 March 2019. Standard errors are clustered at the helpline-week level. We repeat the analysis using data from 1 January up to 24 March in 2019 and 2022, where we define the indicator $Post\,invasion_{t}$ as equal to one for calls on/after 24 February 2022.

## Supplementary Information

Supplementary Information.

## Data Availability

Data were provided by helplines for the sole purpose of this research project, subject to confidentiality agreements. Researchers can obtain (updated) helpline data after signing agreements with the individual helplines. For further information, contact info@nummergegenkummer.de and presse@telefonseelsorge.de. The full data underlying specific parts of the analysis are available from the authors at rafael.lalive@unil.ch upon reasonable request, conditional on permission of the respective helplines. All analyses were conducted using Stata/SE 17.0. Do files and Figure source data are available online at 10.5281/zenodo.7090520. The data on infection rates and policy measures shown in Fig. 1 are available online from the JHU CSSE COVID-19 Dataset^8^, and the Oxford COVID-19 Government Response Tracker^73^.
