# Supplementary material for: Young people’s mental and social distress in times of international crisis: evidence from helpline calls, 2019–2022
Source: Sci Rep. 2023 Jul 22;13:11858. doi: 10.1038/s41598-023-39064-y (PMC10363110; doi:10.1038/s41598-023-39064-y)
Supplement: Supplementary file 1 — Supplementary Information. [file 41598_2023_39064_MOESM1_ESM.pdf]

Supplementary information

Young people's mental and social distress in times of international crisis: Evidence from helpline calls, 2019-2022

July 13, 2023

Marius Brülhart[1,2], Valentin Klotzbücher[3] & Rafael Lalive[1,2,*]

[1] University of Lausanne, Faculty of Business and Economics (HEC Lausanne), Lausanne, Switzerland
[2] CEPR, London, United Kingdom
[3] University of Freiburg, Institute of Economics, Freiburg im Breisgau, Germany
[*] Corresponding author: rafael.lalive@unil.ch

This PDF file includes:

- Supplementary Figures S1–S9
- Supplementary Tables S1–S7

Other supporting materials for this manuscript include the following:

- SIData.xlsx: Figure source data, including numerical estimates
- Stata Do-files

Files are available online at https://doi.org/10.5281/zenodo.7090520

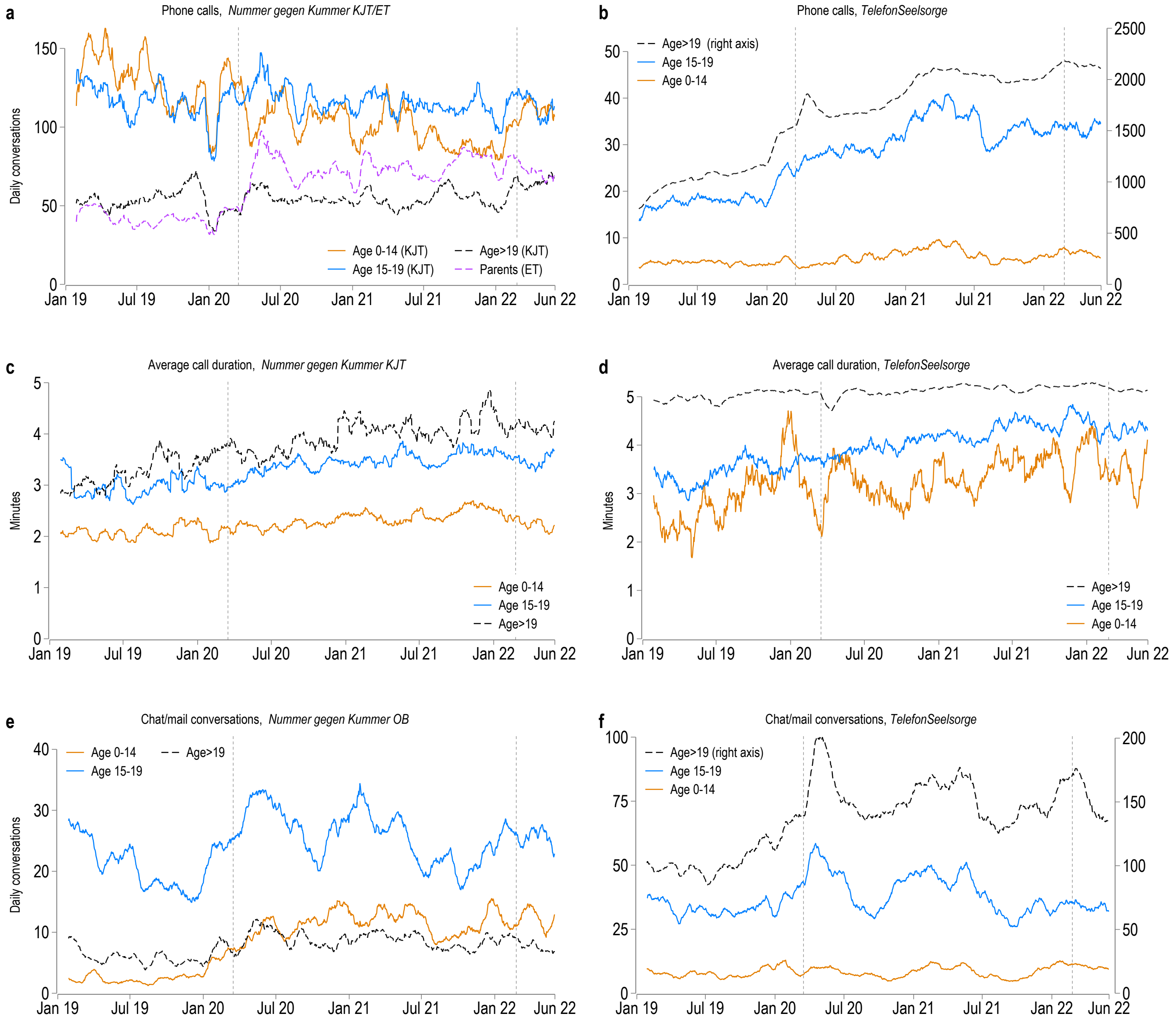

Supplementary Fig. S1: Call volumes across helpline services. The top panels show the development of phone calls per day by age group, separately for phone calls with, **A**, the *Nummer gegen Kummer* child/youth helpline and the parent helpline, and, **B**, the *TelefonSeelsorge* general-purpose helpline. The average duration of calls with the two helplines are shown in panels **C** and **D**. Panels **E** and **F** show the number of chat/mail contacts at the two services. All panels show 28-day moving averages. Vertical dashed lines mark, respectively, the introduction of lockdown measures following the COVID-19 pandemic outbreak on 16 March 2020 and the Russian invasion of Ukraine on 24 February 2022.

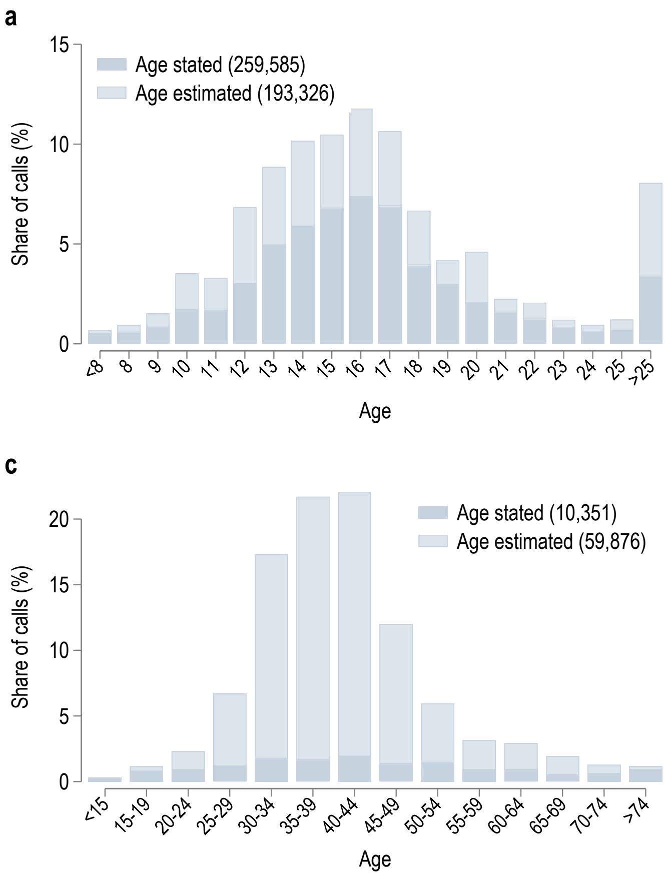

Supplementary Fig. S2: Age distribution of callers Share of contacts for different age groups across the threee helplines: **A**, NgK child/youth helpline (KJT phone and OB chat/mail), **B**, TS (phone and chat/mail) , **C**, NgK parent helpline. Dark shading indicates the share of calls for which callers explicitly stated their age during the conversation, light grey for contacts where caller age had to be inferred by counselors.

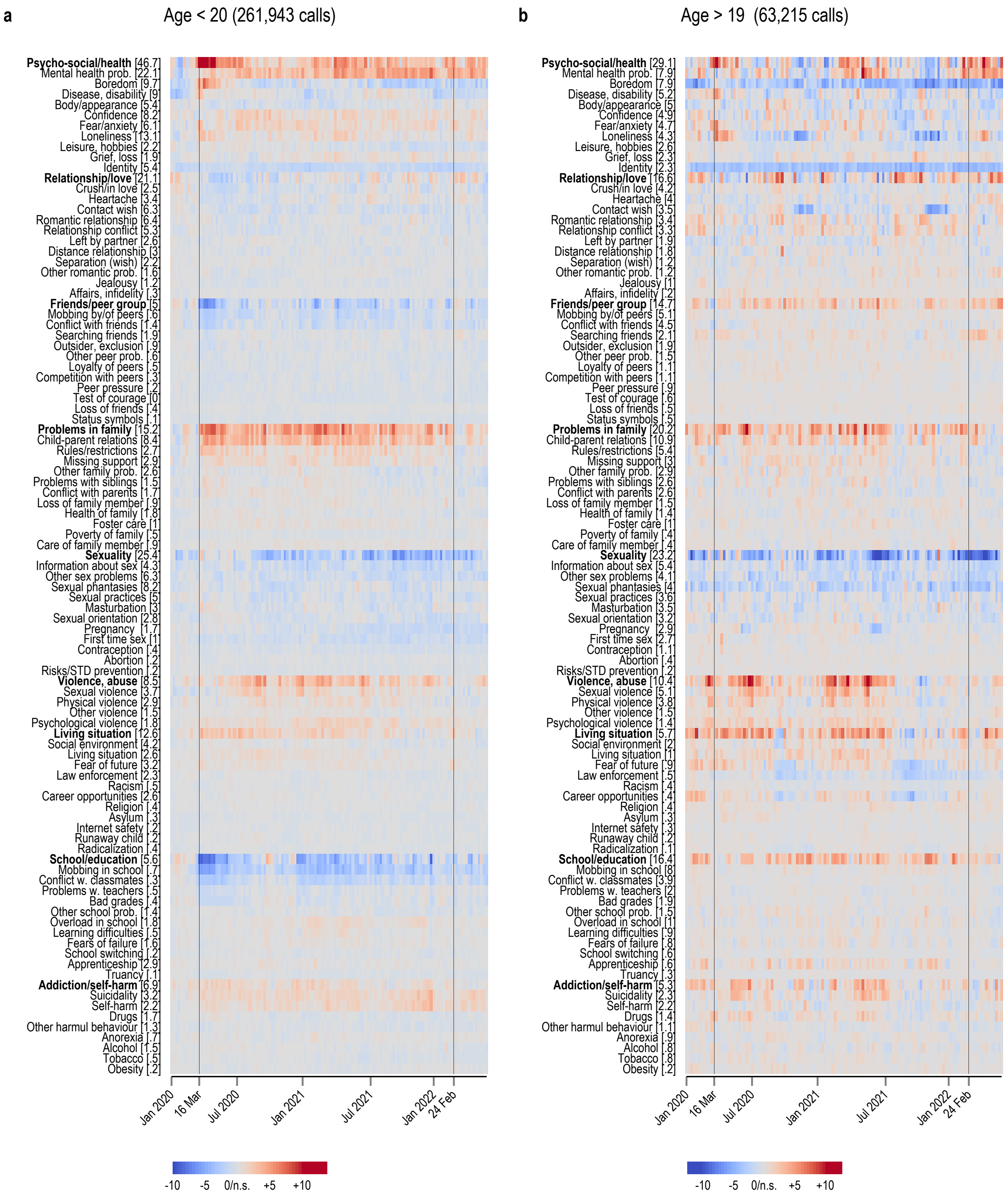

Supplementary Fig. S3: Conversation topics over time, NgK child/youth helpline. Deviation of relative topic prevalence from 2019 baseline in percentage points, calls with *Nummer gegen Kummer* child/youth helpline. Each row presents coefficient estimates for week indicators from a linear probability model where the dependent variable is set to one for calls related to a topic and zero otherwise; numbers in brackets report the share of calls in 2019 related to each topic in percent. Bold topics refer to broad categories, indicating calls that relate to at least one of the fine topics listed below respectively. See Methods, equation 2. Conversations with, **A**, children and adolescents up to age 19, and, **B**, adult callers of age 20 or older. Statistically significant (P-value<0.05) increases are shown in red, decreases in blue, insignificant coefficients as zero/in gray. Vertical black lines mark, respectively, the introduction of lockdown measures following the COVID-19 pandemic outbreak on 16 March 2020 and the Russian invasion of Ukraine on 24 February 2022.

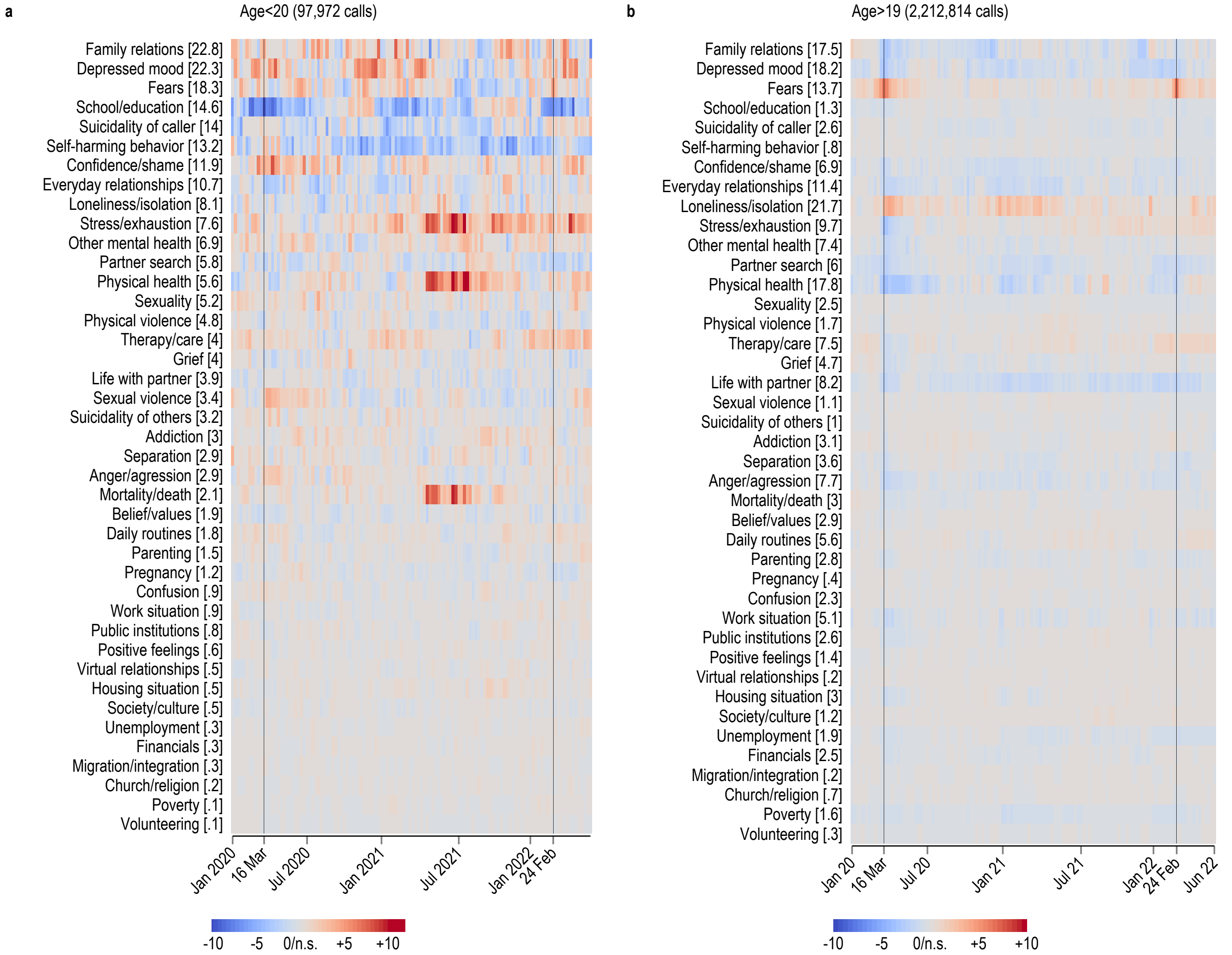

Supplementary Fig. S4: Conversation topics over time, TelefonSeelsorge. Deviation of relative topic prevalence from 2019 baseline in percentage points, calls with *TelefonSeelsorge* general-purpose helpline. Each row presents coefficient estimates for week indicators from a linear probability model where the dependent variable is set to one for calls related to a topic and zero otherwise; numbers in brackets report the share of calls in 2019 related to each topic in percent. See Methods, equation 2. Conversations with, **A**, children and adolescents up to age 19, and, **B**, adult callers aged 20 or older. Statistically significant (P-value<0.05) increases are shown in red, decreases in blue, insignificant coefficients as zero/in gray. Vertical black lines mark, respectively, the introduction of lockdown measures following the COVID-19 pandemic outbreak on 16 March 2020 and the Russian invasion of Ukraine on 24 February 2022.

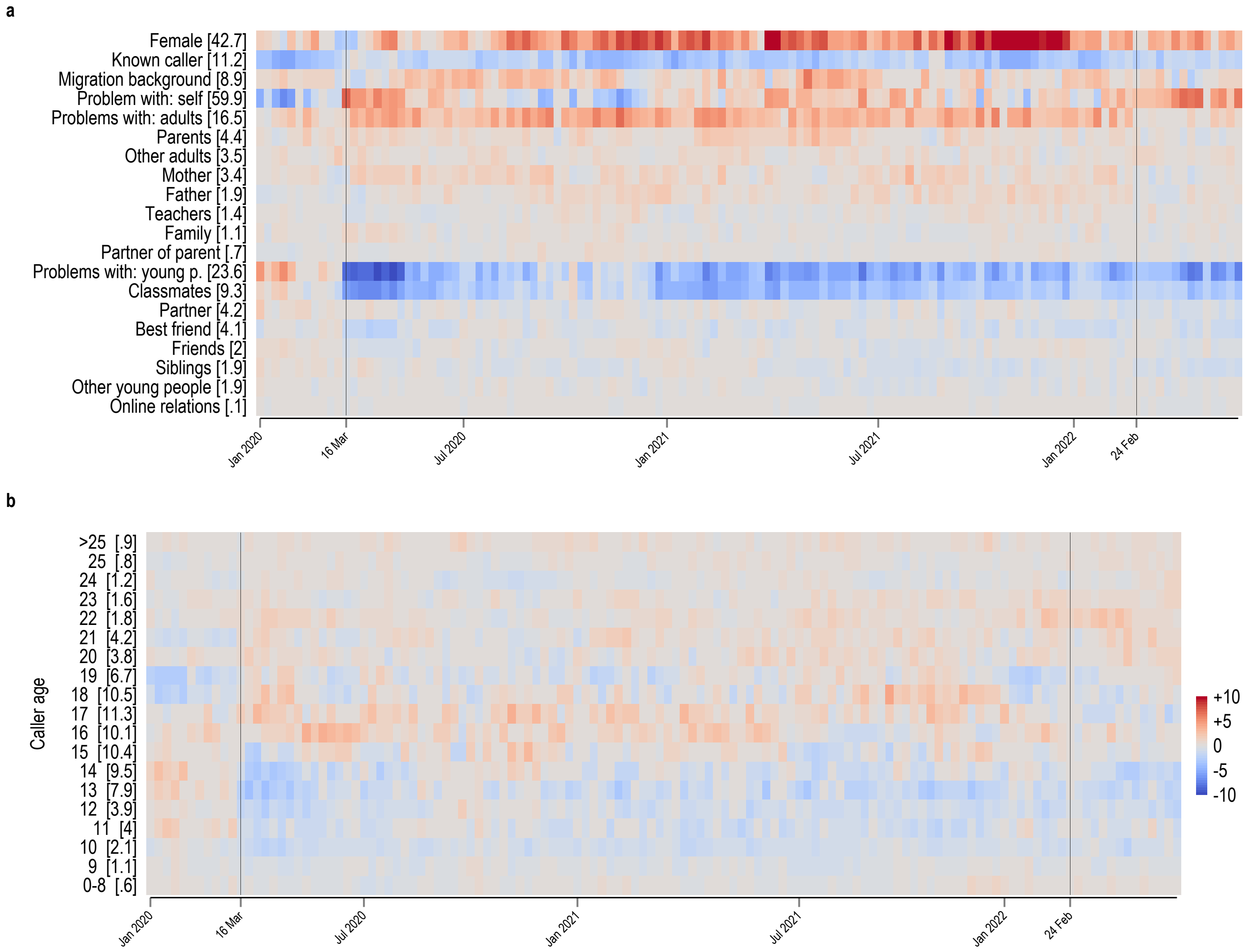

Supplementary Fig. S5: Caller characteristics over time, NgK child/youth helpline. Deviation of relative share from 2019 baseline in percentage points, calls with *Nummer gegen Kummer* children and youth helpline. Each row presents coefficient estimates for week indicators from a linear probability model where the dependent variable is set to one for calls related to a topic and zero otherwise; numbers in brackets report the share of calls in 2019 related to each topic in percent. See Methods, equation 2. Sample size varies depending on availability, see Table S2. Conversations with, **A**, children and adolescents up to age 19, and, **B**, adult callers aged 20 or older. Statistically significant (P-value<0.05) increases are shown in red, decreases in blue, insignificant coefficients as zero/in gray. Vertical black lines mark, respectively, the introduction of lockdown measures following the COVID-19 pandemic outbreak on 16 March 2020 and the Russian invasion of Ukraine on 24 February 2022.

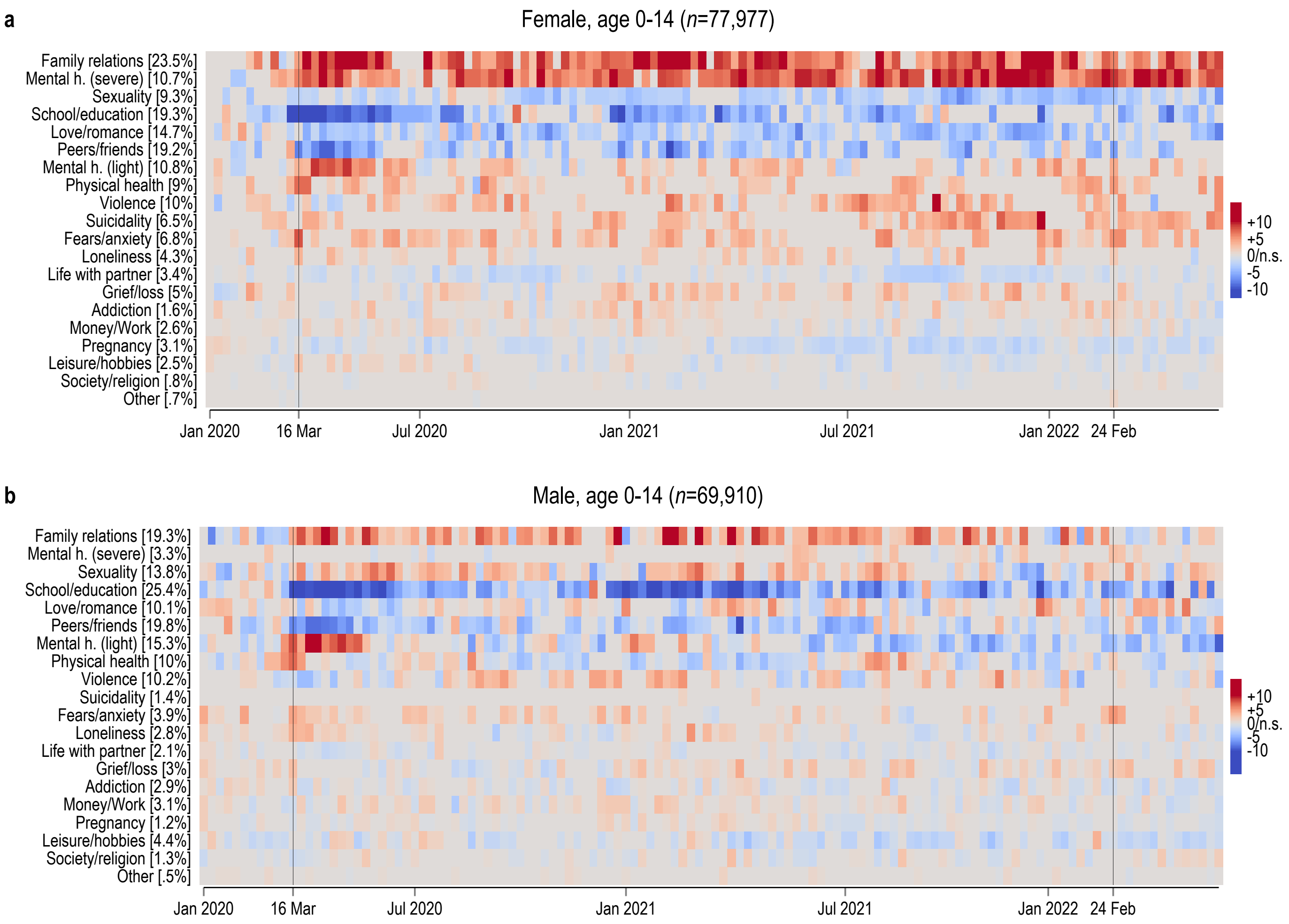

Supplementary Fig. S6: Conversation topics by caller sex: children. Deviation of relative topic prevalence from 2019 baseline in percentage points, calls with *Nummer gegen Kummer* child/youth helpline and *TelefonSeelsorge* general-purpose helpline. Each row presents coefficient estimates for week indicators from a linear probability model where the dependent variable is set to one for calls related to a topic and zero otherwise; numbers in brackets report the share of calls in 2019 related to each topic in percent. Separate models for, **A**, female and, **B**, male children up to age 14. See Methods, equation 2. Vertical black lines mark, respectively, the introduction of lockdown measures following the COVID-19 pandemic outbreak on 16 March 2020 and the Russian invasion of Ukraine on 24 February 2022.

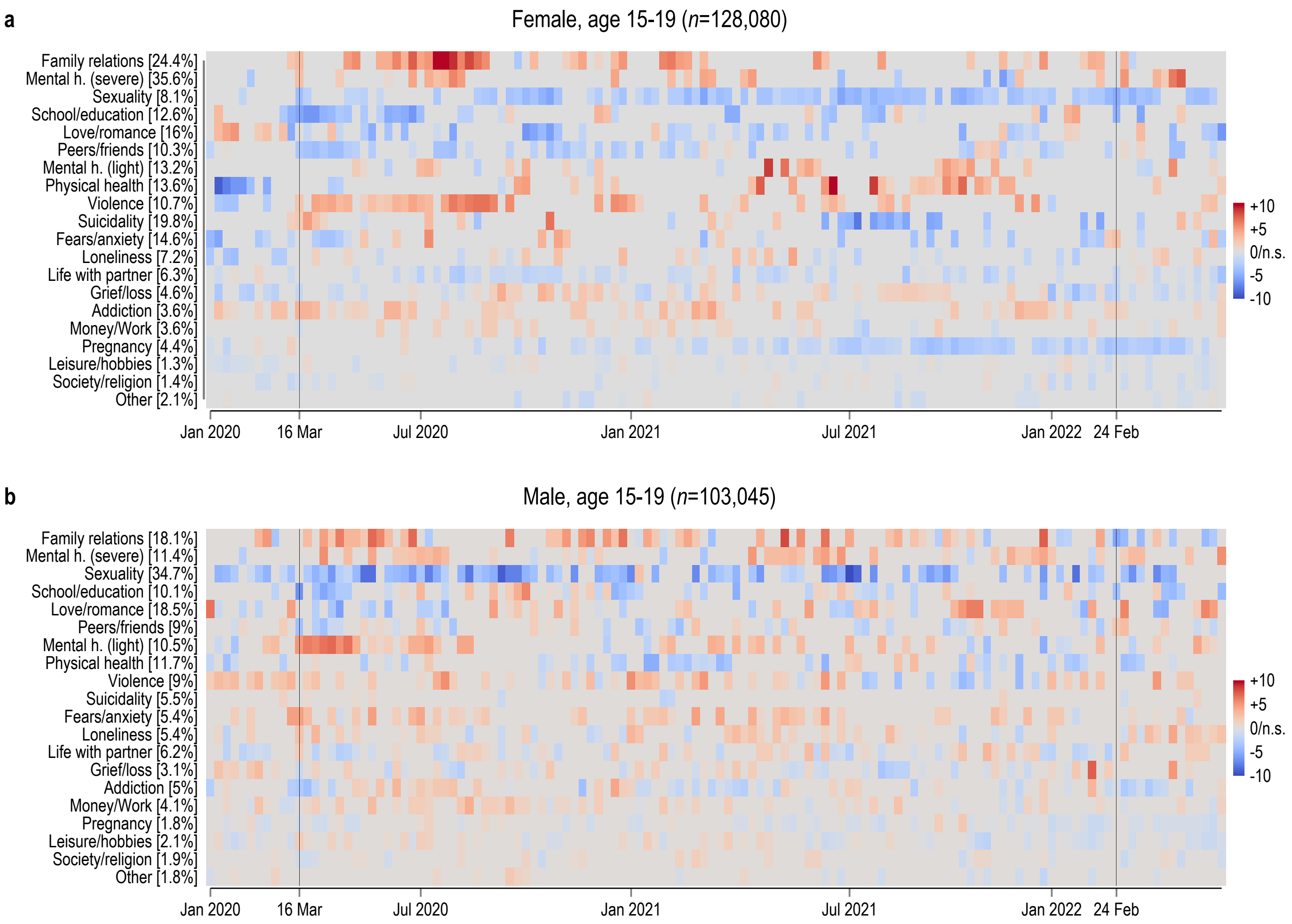

Supplementary Fig. S7: Conversation topics by caller sex: adolescents. Deviation of relative topic prevalence from 2019 baseline in percentage points, calls with *Nummer gegen Kummer* child/youth helpline and *TelefonSeelsorge* general-purpose helpline. Each row presents coefficient estimates for week indicators from a linear probability model where the dependent variable is set to one for calls related to a topic and zero otherwise; numbers in brackets report the share of calls in 2019 related to each topic in percent. Separate models for, **A**, female and **B**, male adolescents aged 15–19. See Methods, equation 2. Vertical black lines mark, respectively, the introduction of lockdown measures following the COVID-19 pandemic outbreak on 16 March 2020 and the Russian invasion of Ukraine on 24 February 2022.

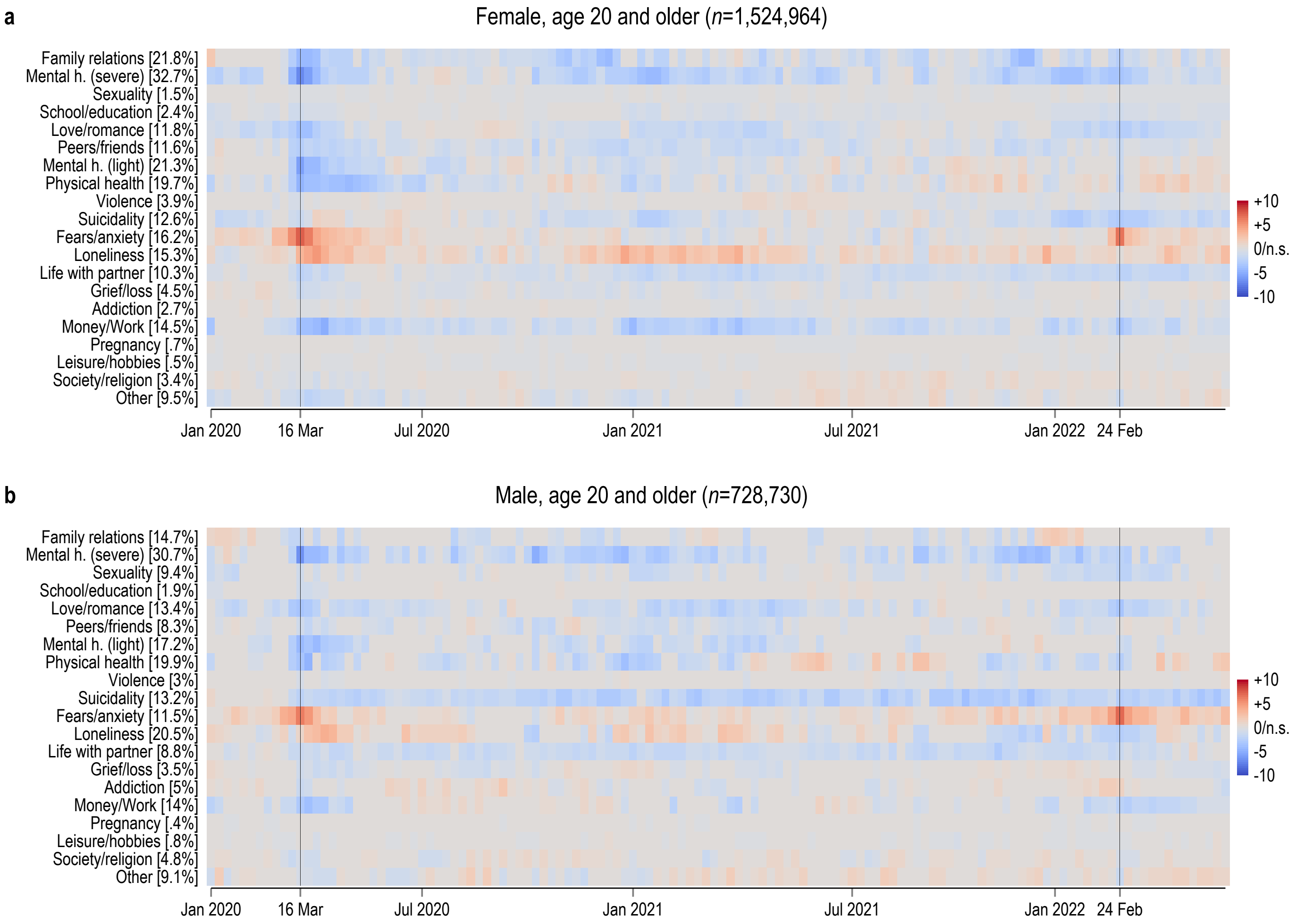

Supplementary Fig. S8: Conversation topics by caller sex: adults. Deviation of relative topic prevalence from 2019 baseline in percentage points, calls with *Nummer gegen Kummer* child/youth helpline and *TelefonSeelsorge*. Each row presents coefficient estimates for week indicators from a linear probability model where the dependent variable is set to one for calls related to a topic and zero otherwise; numbers in brackets report the share of calls in 2019 related to each topic in percent. Separate models for, **A**, female and, **B**, male callers aged 20 and older. See Methods, equation 2. Vertical black lines mark, respectively, the introduction of lockdown measures following the COVID-19 pandemic outbreak on 16 March 2020 and the Russian invasion of Ukraine on 24 February 2022.

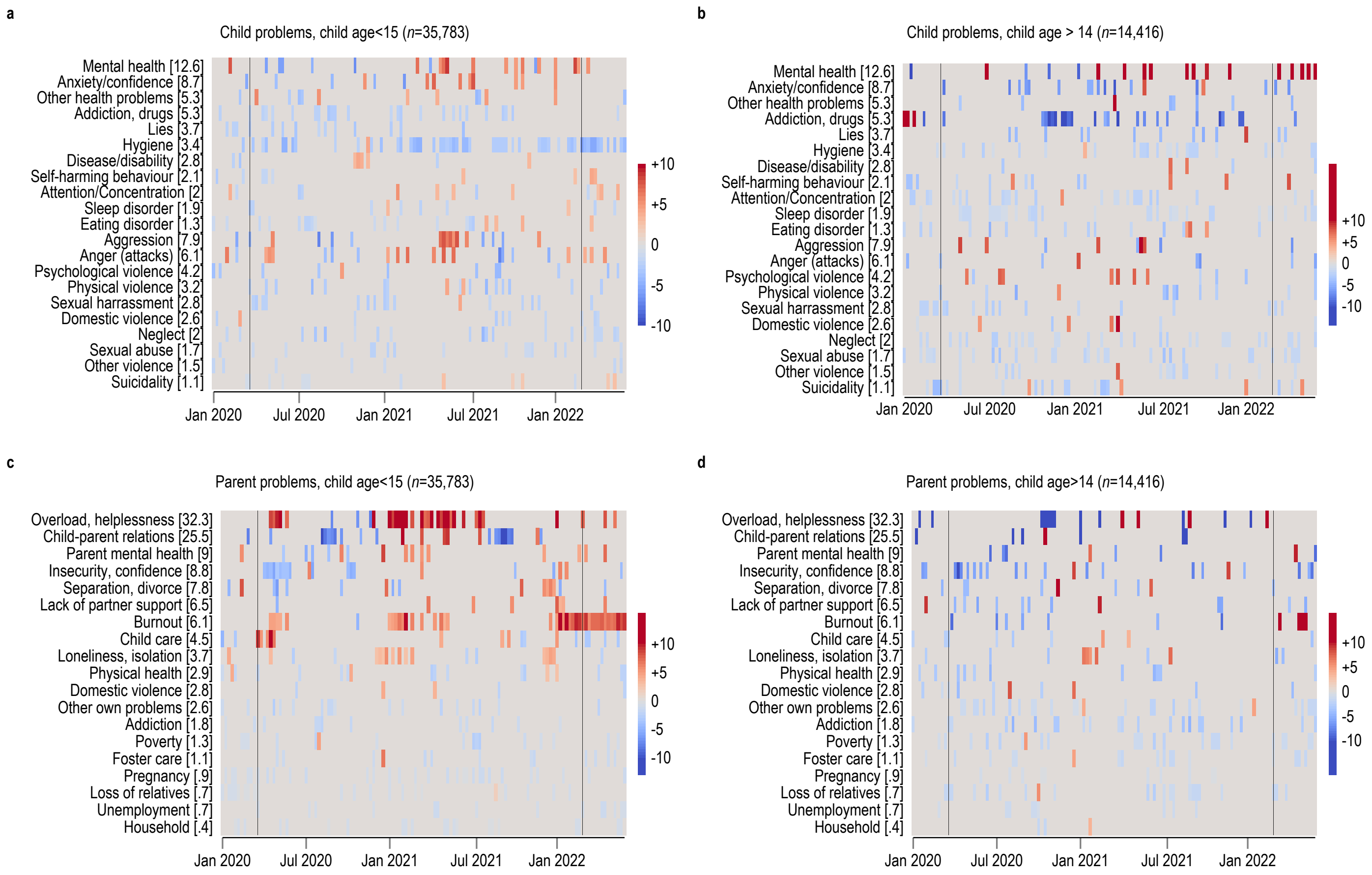

Supplementary Fig. S9: Parent helpline, topics by child age. Deviation of relative topic prevalence from 2019 baseline in percentage points, calls with *Nummer gegen Kummer* parent helpline. See Methods, equation 2. The upper panels show trends for problems related to children, separately for, **A**, callers referring to children and, **B**, callers referring to adolescents. Note that calls can relate to both groups and the two samples are non-exclusive. The bottom panels show separate trends in other caller problems, again for, **C**, children and, **D**, adolescents. Statistically significant (P-value<0.05) increases are shown in red, decreases in blue, insignificant coefficients as zero/in gray. Vertical black lines mark, respectively, the introduction of lockdown measures following the COVID-19 pandemic outbreak on 16 March 2020 and the Russian invasion of Ukraine on 24 February 2022.

**A** NgK children/youth line	Share	Sum	n
Female caller	0.464	170,790	368,123
Male caller	0.527	193,888	368,123
Phone	0.865	325,158	375,798
Chat	0.107	40,258	375,798
Mail	0.028	10,382	375,798
Caller age <15	0.370	138,869	375,638
Caller age 15–19	0.436	163,894	375,638
Caller age >19	0.194	72,875	375,638
**B** TelefonSeelsorge	Share	Sum	n
Female caller	0.684	1,700,302	2,484,971
Male caller	0.316	784,669	2,484,971
Phone	0.904	2,306,742	2,550,473
Chat	0.040	103,216	2,550,473
Mail	0.055	140,515	2,550,473
Caller age <15	0.008	17,487	2,322,943
Caller age 15–19	0.035	80,988	2,322,943
Caller age >19	0.958	2,224,468	2,322,943
**C** Combined dataset	Share	Sum	n
Female caller	0.656	1,871,092	2,853,094
Male caller	0.343	978,557	2,853,094
Phone	0.899	2,631,900	2,926,271
Chat	0.049	143,474	2,926,271
Mail	0.052	150,897	2,926,271
Caller age <15	0.058	156,356	2,698,581
Caller age 15–19	0.091	244,882	2,698,581
Caller age >19	0.851	2,297,343	2,698,581
NgK children/youth line	0.128	375,798	2,926,271
TelefonSeelsorge	0.872	2,550,473	2,926,271

Supplementary Table S1: Summary Statistics, combined dataset. Calls with, **A**, the NgK child/youth helpline, **B**, TelefonSeelsorge general-purpose helpline, and, **C**, the combined dataset used in the main analysis. Columns show the respective share and number of calls, along with the overall number of calls for which information is available.

	Share	Sum	n
Female	0.464	170,830	368,169
Male	0.527	193,894	368,169
Diverse	0.021	7,433	362,251
Phone call	0.865	325,158	375,844
Chat contact	0.107	40,258	375,844
Mail contact	0.028	10,428	375,844
Migration background	0.100	21,284	213,646
Known caller	0.092	34,403	375,844
Worried about another person	0.068	22,656	335,586
Problems with self	0.605	196,840	325,158
Problems with young people	0.208	67,571	325,158
siblings	0.017	5,630	325,158
best friend	0.038	12,418	325,158
friends	0.019	6,269	325,158
partner	0.041	13,392	325,158
classmates	0.072	23,391	325,158
online relations	0.002	648	325,158
other young people	0.018	5,823	325,158
Problems with adults	0.187	60,747	325,158
with parents	0.049	16,012	325,158
with father	0.023	7,598	325,158
with mother	0.040	13,010	325,158
with partner of parent	0.008	2,645	325,158
in family	0.012	4,014	325,158
with teachers	0.015	4,913	325,158
with other adults	0.039	12,555	325,158

Supplementary Table S2: Summary Statistics, NgK child/youth helpline. Conversations with the NgK child/youth helpline. Columns show the respective share and number of calls, along with the overall number of conversations for which information is available.

	Share	Sum	n
Female	0.687	1,576,477	2,294,819
Male	0.313	718,342	2,294,819
Other gender	0.003	6,769	2,301,588
Phone contacts	0.902	2,081,504	2,308,411
Chat contacts	0.040	92,619	2,308,411
Mail contacts	0.058	134,288	2,308,411
Living alone	0.658	1,384,270	2,103,129
in institution	0.049	103,102	2,103,129
with family	0.135	283,074	2,103,129
with partner	0.134	281,239	2,103,129
in shared accom.	0.024	51,444	2,103,129
Working	0.277	521,455	1,884,109
In education	0.083	155,643	1,884,109
Unemployed, searching	0.059	111,894	1,884,109
Unempl., not searching	0.060	113,923	1,884,109
Retired	0.254	478,544	1,884,109
Disability	0.267	502,650	1,884,109
Known psychol. diagnosis	0.345	797,147	2,308,409
Suicidal thoughts	0.082	188,983	2,308,405
Suicide plans	0.013	29,969	2,308,405
Suicide attempt	0.012	27,438	2,308,405
Suicidality of others	0.012	27,844	2,308,405

Supplementary Table S3: Summary Statistics, TelefonSeelsorge. Calls with TelefonSeelsorge general-purpose helpline. Columns show the respective share and number of calls, along with the overall number of calls for which information is available.

	Share	Sum	n
Female caller	0.749	41,782	55,787
Male caller	0.251	14,005	55,787
First-time caller	0.845	29,212	34,566
Repeat caller	0.155	5,354	34,566
Habitual caller	0.028	966	34,566
Migration background	0.120	5,713	47,515
Financial problems	0.258	6,645	25,751
Married/partner	0.554	25,070	45,252
Single	0.133	6,003	45,252
Separated	0.182	8,244	45,252
Divorced	0.105	4,736	45,252
Widowed	0.026	1,199	45,252
Parents	0.852	45,256	53,093
Foster parents	0.005	241	53,093
Partners of parent	0.032	1,711	53,093
Grandparents	0.046	2,452	53,093
Other relatives	0.027	1,446	53,093
Friends	0.020	1,074	53,093
Neighbors	0.010	536	53,093
Teachers	0.007	377	53,093
Children living w. caller	0.685	38,477	56,150
Children not living w. caller	0.260	14,585	56,150
Unknown	0.055	3,088	56,150
Referring to children (age<15))	0.735	35,783	48,686
adolescents (age>14)	0.296	14,416	48,686
male children/adolescents	0.539	28,847	53,513
female children/adolescents	0.532	28,625	53,759

Supplementary Table S4: Summary Statistics, NgK parent helpline. Calls with the NgK parent helpline. Columns show the respective share and number of calls, along with the overall number of calls for which information is available.

	Children, age<15				Adolescents, age 15–19			
	2019	2020	2021	2022	2019	2020	2021	2022
Contacts	47,796	47,228	41,724	19,760	64,937	75,718	74,010	30,975
Duration (min)	10.09	10.72	12.12	11.29	14.60	16.66	18.51	18.09
Phone call (%)	92.64	85.68	82.32	82.27	69.73	66.53	69.20	69.71
Chat (%)	2.02	2.91	3.15	3.34	6.82	9.22	8.50	8.96
Mail (%)	3.97	3.99	5.11	6.11	14.20	14.19	12.91	11.37
Female (%)	49.95	50.68	56.15	54.16	52.12	55.45	58.00	56.86
Male (%)	48.66	47.82	42.48	44.36	47.52	44.22	41.66	42.73

	Adults. age>19				Age unknown			
	2019	2020	2021	2022	2019	2020	2021	2022
Contacts	429,238	692,577	819,395	384,667	40,244	69,587	81,183	39,833
Duration (min)	24.64	25.15	25.88	25.66	12.19	12.73	13.66	13.80
Phone call (%)	90.84	91.50	92.92	93.14	92.12	92.20	93.97	94.38
Chat (%)	3.22	3.48	2.85	2.94	4.55	5.10	3.97	3.70
Mail (%)	5.49	4.60	3.87	3.63	3.32	2.49	2.07	1.92
Female (%)	66.80	67.62	68.20	67.24	63.93	64.55	65.38	65.26
Male (%)	33.18	32.37	31.78	32.75	36.07	35.45	34.62	34.74

	Overall sample			
	2019	2020	2021	2022
Contacts	582.215	885.110	1.016.312	475.235
Duration (min)	21,62	22,86	23,95	23,72
Phone call (%)	88,72	89,11	90,84	91,27
Chat (%)	3,57	4,01	3,32	3,37
Mail (%)	6,08	5,13	4,36	4,03
Female (%)	63,57	65,48	66,79	65,89
Male (%)	36,25	34,40	33,12	34,01

Supplementary Table S5: Call characteristics over time, combined dataset. Calls with the NgK child/youth helpline and TelefonSeelsorge general-purpose helpline, by age of caller and year.

Topic	*Nummer gegen Kummer* child and youth helpline	*TelefonSeelsorge*
Family relations	Child-parent relations, Conflict between parents, Care of family member, Foster care, Missing support, Problems with siblings, Rules/restrictions, Runaway child, Other family problems	Family relations, Parenting
Mental health, severe	Self-harming behaviour, Mental health problems	Self-harming behavior, Depressed mood, Other mental health problems
Sexuality	Contraception, First time sex, Sexual orientation, Masturbation, Sexual practices, Risks/STD prevention, Sexual phantasies, Information about sex, Identity, Other sex problems	Sexuality
School/education	Mobbing in school, Truancy, School switching, Learning difficulties, Fears of failure, Apprenticeship, Overload, Bad grades, Problems w. teachers, Conflict w. classmates, Other school problems	School, education
Love/romance	Affairs/infidelity, Jealousy, Heartache, Crush/in love, Romantic relationship, Contact wish, Separation, Left by partner, Other romantic problems	Partner search, Separation/divorce
Peers/friends	Status symbols, Loss of friends, Test of courage, Peer pressure, Competition with peers, Loyalty of peers, Outsider, exclusion, Searching friends, Conflict with friends, Mobbing by/of peers, Social environment, Other peer problems	Everyday relationships
Mental health, moderate	Boredom, Confidence	Stress/emotional exhaustion, Confusion, Confidence/shame, Anger, aggression
Physical health	Disease/disability, Obesity, Anorexia, Health of family member, Body/appearance	Physical health, Therapy/care
Violence	Physical violence; Sexual violence; Psychological violence; Other violence	Physical violence, Sexual violence
Suicidality	Suicide	Suicidality of caller, Suicidality of others
Fear/anxiety	Fear/anxiety, Fear of future	Fears
Loneliness/isolation	Loneliness	Loneliness/isolation
Life with partner	Relationship conflict, Distance relationship	Life with partner
Addiction	Tobacco, Alcohol/drugs	Addiction
Grief/loss	Grief/loss, Loss of family member	Grief, Mortality/death
Money/Work	Career opportunities, Living situation, Poverty in family	Unemployment, Financials, Work situation, Poverty, Housing situation
Pregnancy	Pregnancy, Abortion	Pregnancy
Leisure/hobbies	Leisure/hobbies, Internet safety	Volunteering, Virtual relationships
Society/religion	Religion, Asylum, Racism, Radicalization	Society/culture, Belief/values, Church/religion, Migration/integration
Other	Law enforcement	Daily routines, Positive feelings, Public institutions

Supplementary Table S6: Consolidation of conversation topics. Classification of conversation topics from helpline-specific (NgK child/youth helpline and TS general-purpose helpline) categories into 20 consolidated topics.

Topic	*Nummer gegen Kummer* children and youth helpline	*TelefonSeelsorge*
Family relations	Verbote, Regeln, Meinungen Konflikte der Eltern, Scheidung;Geschwisterproblematik; Benachteiligung, fehlende Unterstützung; Pflege, Unterstützung Angehöriger; Fremdunterbringung; Ausreißer, Straßenkinder	Familiäre Beziehungen; Elternschaft, Erziehung
Mental health; severe	Psychische Probleme; Selbstverletzung	Depressive Stimmung; Selbstverletzendes Verhalten; Sonstiges seelisches Befindenden
Sexuality	Verhütung; "das erste Mal"; Infos zu Körper, Entwicklung, Sexualität; Selbstbefriedigung; sexuelle Praktiken; sex. Phantasien; sexuelle Orientierung (z.B. Homosexualität); Risiken (AIDS, Krankheiten, Prävention); Identität (Transgender)	Sexualität
School/education	Schwänzen, Schulverweigerung Lernschwierigkeiten; Schulwechsel; Versagensängste; Probleme in Ausbildung, Beruf; Überforderung, Leistungsdruck; schlechte Noten, Zeugnis; Probleme mit Lehrern; Streit, ärger mit Mitschülern; Spott, Ausgrenzung, Mobbing	Schule, Ausbildung
Love/romance	Kontaktwunsch; Schwärmen, Verliebtheit; Gestaltung einer Partnerschaft; Liebeskummer; Eifersucht; Untreue; Trennung (-wunsch); ist verlassen worden	Partnersuche, Partnerwahl; Trennung
Peers/friends	Spott, Ausgrenzung durch Gleichaltrige; Streit, ärger Freundeskreis; Wunsch nach Freund(en); Außenseiter; Loyalitätsprobleme; Verlust Freundeskreis (Umzug etc.); Gruppendruck; Statussymbole; Konkurrenz, Eifersucht; Mutprobe	Alltagsbeziehungen, Nachbarn, Freunde
Mental health; moderate	Selbstvertrauen; Langeweile	Stress, emotionale Erschöpfung; Verwirrtheitszustände; Selbstbild, Selbstwert, Scham; Ärger; Agression
Physical health	Krankheit, Behinderung; Körper, Aussehen; Sucht, psychische Probleme der Eltern, körp. Erkrankungen Angehörige	Körperl. Befinden, Beschwerden; Betreuung, Pflege, Therapie, Behandlung
Violence	körperliche Gewalt; Sexueller Missbrauch; sexuelle übergriffe; psychische Gewalt; Vernachlässigung; Gewaltandrohung; Erpressung; Sexuelle Grenzverletzung; Zeuge häuslicher Gewalt; Opfer häuslicher Gewalt	Körperliche Gewalt, seelische Gewalt; Sexualisierte Gewalt
Suicidality	Suizidgedanken, -versuch	Suizidalität des Ratsuchenden; Suizidalität Anderer; Suizidgedanken; Suizidabsichten; Suizidversuch
Fears/anxiety	Furcht, Angst; Zukunftsängste	Ängste
Loneliness	Einsamkeit, Isolation	Einsamkeit; Isolation
Life with partner	Beziehungskonflikte; Fern-, Urlaubsbeziehung	Leben in Partnerschaft
Addiction	Drogen, Drogensucht; speziell Tabak; speziell Alkohol	Sucht
Grief/loss	Trauer, Verlust, Tod; Trauer, Verlust in Familie	Trauer; Sterben, Tod
Money/Work	Finanzfragen, Erbschaft, Unterhalt; Armutsproblematik; Bildungs- und Arbeitsmöglichkeiten; Wohnung, Wohnumfeld; Wohnverhältnisse, Wohnumfeld	Arbeitslosigkeit, Arbeitssuche; Arbeitssituation; Armut
Pregnancy	Schwangerschaft;Schwangerschaftsabbruch	Schwangerschaft; Kinderwunsch
Leisure, hobbies	Freizeit(-gestaltung), Hobby; Sicherheit im Internet	Ehrenamtl. Tätigkeit; Virtuelle Beziehungen
Society/religion	Religion; Asyl- und Flüchtlingsthematik; Rassismus, Fremdenfeindlichkeit; Radikalismus, Extremismus	Gesellschaft und Kultur; Kirchen, Glaubensgemeinschaft; Sinn, Glaube, Werte; Migration, Integration
Other	Konflikte mit Gesetz, Behörden	Alltagsgestaltung; Positives Befinden, Freude, Dank; Kontakt mit öffentl. Einrichtungen

Supplementary Table S7: Consolidation of topics, German original. Classification of conversation topics from helpline-specific (NgK child/youth helpline and TS general-purpose helpline) categories into 20 consolidated topics. German titles as stated in reporting software used by operators and helpline statistics.
